# Mapping the Research on Health Policy and Services in the Last Decade (2009–2018): A Bibliometric Analysis

**DOI:** 10.3389/fpubh.2022.773668

**Published:** 2022-04-27

**Authors:** Linyan Zhao, Yang Zhao, Jian Du, Allissa Desloge, Zhiyong Hu, Gaofang Cao

**Affiliations:** ^1^School of Public Health and Management, Binzhou Medical University, Yantai, China; ^2^The George Institute for Global Health, University of New South Wales, Sydney, NSW, Australia; ^3^The George Institute for Global Health at Peking University Health Science Center, Beijing, China; ^4^School of Public Health, University of Illinois Chicago, Chicago, IL, United States; ^5^National Institute of Health Data Science, Peking University, Beijing, China; ^6^Institute of Medical Information, Chinese Academy of Medical Sciences, Beijing, China

**Keywords:** health policy, health services, research trends, research hotpots, bibliometric analysis

## Abstract

**Background:**

Health policy and services is a continuously evolving field of research that can inform prevention and control efforts for a variety of health conditions. The “Healthy China” strategy reflects the demand to formulate health policy that suits China's national needs and goals. Applying bibliometric analysis to grasp the general situation of health policy and services research globally will be conducive to informing China's designated health plans and initiatives.

**Method:**

A bibliometric analysis of 58,065 articles on “Health Policy and Services” topics was conducted. The document type was restricted to journal articles that were published in the Web of Science database between the time parameter of January 1, 2009 to December 31, 2018. Data was collected on indicators such as the annual number of publications in the field of health policy and services, the country where the publication is issued, the publication organization, the source journal, the frequency of citations, research hotspots, and academic areas.

**Results:**

The overall number of articles published in Web of Science on health policy and services research has increased over time. The United States has the largest number of articles in the field. The institution with the highest number of citations in the field is Harvard University and the journal with the most published articles in the field is Health Affairs. Research hotspots in the health policy and services field include topics such as “HIV Infections,” “Primary Health Care,” “Delivery of Health Care,” and “Health Services Accessibility.”

**Conclusion:**

Experts in the field of health policy and services globally are dedicated to researching the most effective ways to improve people's health and living standards. There is a certain gap in the depth of health policy and services research between China and developed countries and regions such as Europe or America. China must learn from foreign experience to conduct meaningful and informative research that can aid in the formulation of multi-dimensional health policies in specific areas such as environmental infectious diseases, where attention is needed in areas beyond the medical and health system.

## Introduction

Health policy and services is a broad research field which covers resources on healthcare systems, including healthcare provision and management, financial analysis, healthcare ethics, health policy, and quality of care. It often intersects with other areas to conduct research. The field of health policy and services research continues to play a key role in public health prevention and control efforts and is an important part of the medical and health system framework. In order to understand the scope of health policy and services research around the world, a bibliometric analysis is needed. Through examining the annual number of publications in the field of health policy and services, the country where the publication is issued, the publication organization, the source journal, the frequency of citations, research hotspots, and academic areas in China and abroad will provide sufficient evidence for an objective comparative analysis of the development of the health policy and services field. In general, the application of bibliometric analysis methods to study health policy and services can provide an overview of research in the field and identify research hotspots and research distribution, which is of great significance for the targeted formulation of health policies.

In the process of deepening China's health system reform, the government has promulgated a large number of health policy documents covering the entire medical and health field. In 2016, the State Council of the People's Republic of China (CPC) issued the Healthy China 2030 Planning Outline, proposing a strategy for developing a healthy China (1). In the report at the 19th CPC National Congress, Xi Jinping emphasized that people's health is an essential symbol of national prosperity. It is necessary to improve national health policies and provide comprehensive health services to the general public. These policies and regulations not only reflect the changes in China's health system but also embody the development of public health concepts since 2009. The deepening of health system reform, the Healthy China 2030 strategy and the introduction of a new medical reform program have brought health policy and services research into a new era.

Research on health policy and services in China has progressed greatly due to the enhancement of health reform and the development of medical services. Most studies in the field of health policy and services, however, are either based on descriptive qualitative analysis or employ quantitative analyses on very minimal amounts of data. Furthermore, the results of this quantitative research on health policy are rarely reported from the bibliometric perspective. Bibliometric analyses on academic papers related to health policy have been conducted previously. For example, Ahmed and others (2) employed a bibliometric analysis of multimorbidity to identify and analyze publications on multimorbidity, including those that most influenced this field. Bibliometric analysis provides a method for measuring the academic interest in health policies and estimates the impact of research on health services and policies (3).

Most researchers have focused on disease-specific health policies and the impact of data on relevant health policies, but this study uses bibliometrics to conduct comprehensive analyses with a broad perspective within health policy and services. After summarizing and analyzing existing research, we recognize several problems: (1) Most studies lack holistic and coherent health policy research, focusing on biomedicine, microscopy, and diagnosis, especially on specific diseases or branches of health; (2) there is a big gap between China's health policy research and those of foreign countries in terms of the number of articles published and the scope of coverage. The foreign experience can be used to inform health policies in specific areas such as caring for environmental infectious diseases, but overall, the gaps identified need to be addressed in order to adequately inform medical and health system reform.

Based on the research gap between China and foreign countries, this article conducts a quantitative analysis of domestic and foreign health policy and services literature. We aim to analyze research hotpots by using indicators such as the annual number of publications, the country where the publication is issued, the publication organization, the source journal, the frequency of citations, research hotspots, and academic areas to increase the quality of health policy and services research in China, broaden the coverage of health policies, and ensure that a comprehensive, multi-level health policy that meets China's need can be formulated. This would, in turn, provide basic compliance and guidance for China's medical and health services.

## Methods

Bibliometric methods, which are often used in information and library science as scientometrics, were utilized to analyze the data for this study. Bibliometric methods have previously been applied to analyze the descriptive characteristics of various journals, including the trend in the number of publications, document type, publication year, number of pages and impact factor. Through this method, we can more intuitively conduct a thematic analysis, hotspot analysis and research trend analysis of the past 10 years of health policy and services literature.

### Database and Literature Research

We collected data from Web of Science, the largest database available with about 50% more publications than PubMed (4). First, we searched the database for articles published in INCites using the Category Name: “Health Policy & Services.” This Category Name was determined according to the subject category of the core collection of Web of Science. The INCites database gathers data from the seven major index databases in the core collection of Web of Science within the past 30 years, with diversified indicators and rich visualization effects, which can assist scientific research managers in making strategic decisions more efficiently. Concerning the document type, only published journal articles were included in the analysis. Then, a time parameter from January 1, 2009, to December 31, 2018 was applied. December 31, 2018 was selected as the end date of our search timeframe because in 2019, research related to COVID-19 appeared in the Web of Science database for the first time. This worldwide pandemic has caused a surge in related research. This may affect our discovery of other more valuable studies when conducting bibliometric analysis. A10-year research time span can help us better understand the research progress of health policy and services and identify trends in a relatively moderate time. With this in mind, working backwards from the desired end date, the start time of our search timeframe was set to January 1, 2009. Utilizing the above search parameters, 58,065 articles were retrieved and included in the study. In this paper, the bibliometric method is used to conduct a statistical analysis of the literature in the field of health policy and services research in Web of Science from 2009 to 2018. Academic areas, publication year, citation impact, and periodical influencing factors of the retrieval results were analyzed, and bibliometric analysis was performed in the units of the number of papers.

### Bibliometric Analysis

Bibliometric analysis is a quantitative analysis used to examine the production of academic literature over time (5), it has previously been applied to analyze the descriptive characteristics of various journals, including the trend in the number of publications, document type, publication year, number of pages, and impact factor (IF) (6, 7). A prominent advantage of bibliometrics is that it allows researchers to study specific research areas by analyzing citations, co-citations, geographical distribution, and word frequency.

After comparing the advantages and disadvantages of the BibExcle, Statistical Analysis Toolkit for Infometrics (SATI), CiteSpace and VOSviewer software programs, CiteSpace software was finally selected as the best fit given the features of the data set of this study. CiteSpace has the following advantages: (1) it can directly de-duplicate the data on the Web of science platform and can also directly preprocess the data on platforms such as Scopus and Derwent. (2) the Chinese data can also be preprocessed through deduplication and format conversion. (3) the layout and visualization of clustering networks are more intuitive and easier to interpret. (4) From a micro point of view, both co-occurrence network analysis and literature coupling analysis can be performed to help researchers obtain classic literature in related fields, and to assist users in acquiring research topics, inflection points, and future development trends. It also supports hybrid networks. We used literature metrological analysis software CiteSpace to analyze these research papers for indicators including the annual number of publications, the country where the publication is issued, the publication organization, the source journal, the frequency of citations, research hotspots, and academic areas in order to describe the characteristics of research results in the field of health policy and services.

## Results

### Annual Publication Trends

The annual number of papers published over the past decade reports an overall growth trend, which fluctuated slightly from 2015 to 2018, with a decline between 2015 to 2016 and 2017 to 2018. The growth rate of published literature from 2009 to 2015 is relatively fast, while that from 2016 to 2018 is relatively slow. The year with the highest annual growth rate is from 2012 to 2013, during which the total number of papers increased by 808.

**Figure 1:**
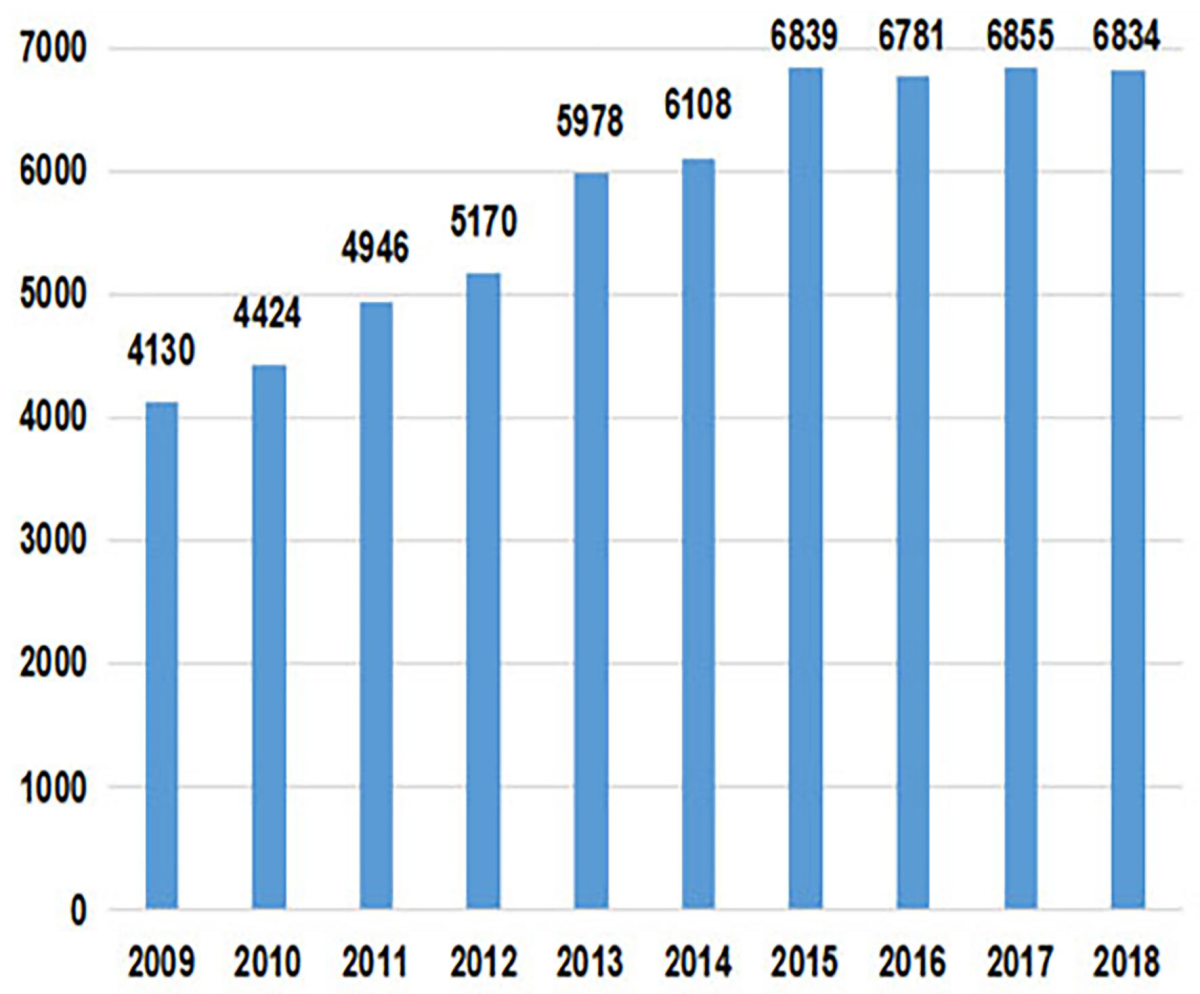
Total number of publications of health policy and services from 2009 to 2018.

**Figure 2:**
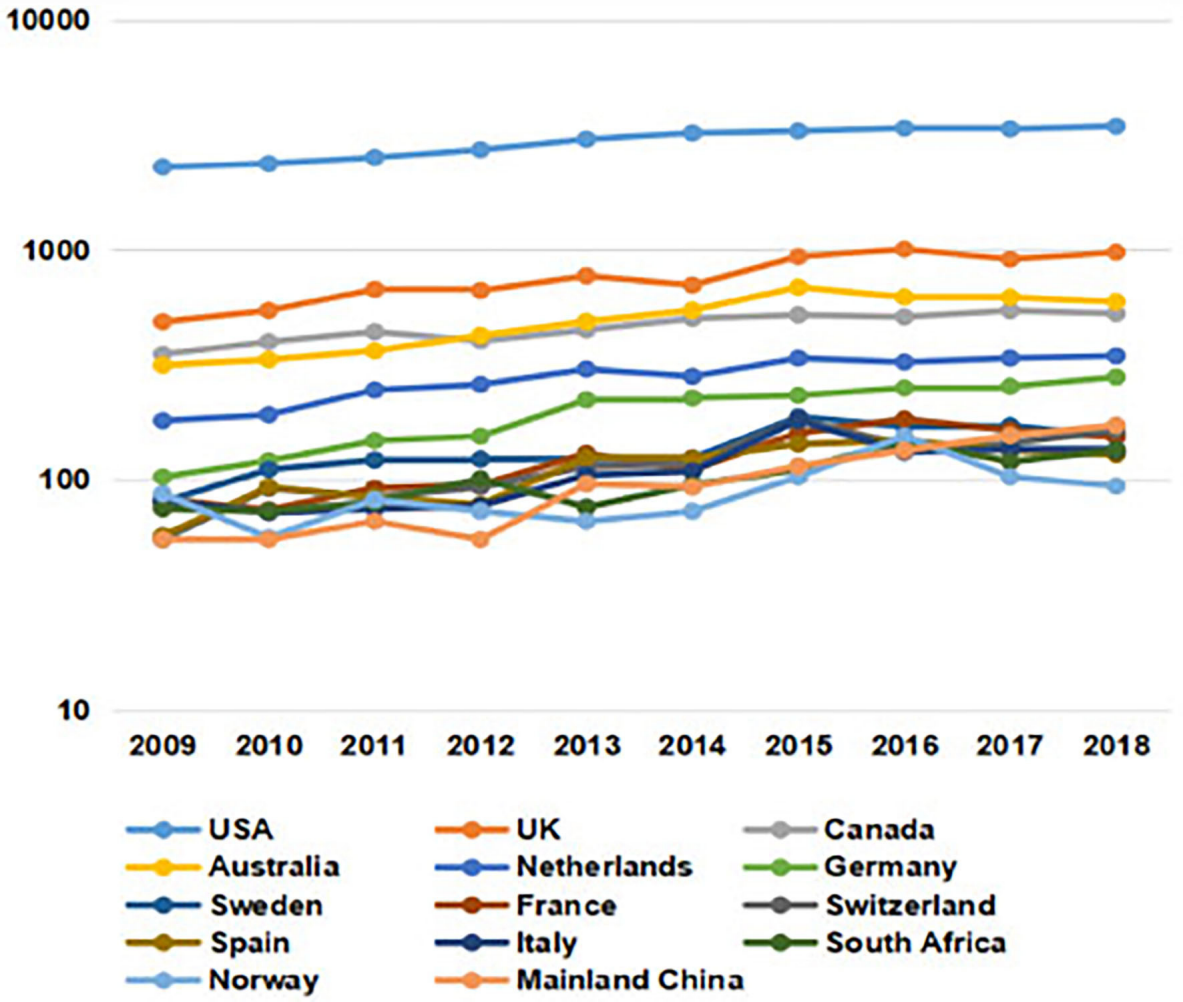
Number of papers of health policy and services from 2009 to 2018 by country.

As is shown in Figures 1, 2, the number of research papers published in the field of health policy and services from 2009 to 2018 shows an overall increasing trend. The United States (US) and the United Kingdom (UK) have always been the top two countries, maintaining a high level of papers. Published papers from Mainland China have also grown rapidly, ranking 6th in 2018.

The research papers we collected data on are mainly from the US, Australia, Sweden, Spain, Norway, the UK, New Zealand, France, Italy, China, Canada, Germany, Switzerland, North Africa, and 14 other countries and regions. The US has the most extensive collection of literature in this field, but its growth has been slow since 2009. Before 2012, there had been relatively little research on health policy and services in mainland China, but it has been slowly increasing since then.

### Top 10 Research Institutions According to Citation Frequency

Table 1 lists the statistical data of scientific research institutions that published papers on health policy and services from 2009 to 2018 and their rankings in descending order of citation frequency. The total number of related literature published by scientific research institutions in various regions is extremely uneven. Among them, institutions in the US have the largest number of papers. Of the top 10 scientific and technological institutions, 8 are from the US, followed by one from the UK and one from Canada.

**Table 1:** Scientific research institutions in the field of health policy and services.

**Rank**	**Research institution**	**Citation frequency**	**Web of science publications**
1	Harvard University	43,578	2,632
2	University of California System	41,546	2,895
3	University of London	27816	2,176
4	Johns Hopkins University	20,699	1,444
5	University of Michigan	19,503	1,124
6	University of Washington	16,901	1,096
7	University of Washington Seattle	16,685	1,075
8	University of Toronto	16,316	1216
9	VA Boston Healthcare System	15,984	1,131
10	University of Pennsylvania	15,810	993

*The ranking is in descending order of citation frequency*.

The top 10 institutions cited have published 15,782 papers, accounting for 27.2% of the total. Harvard University, the University of California System, and the University of London topped the list, with 2,632, 2,895, and 2,176 articles, respectively. Most remaining institutions in the top 10 have published around 1,000 articles each. University of Pennsylvania published <1,000.

The citation frequency ranking of published articles by the top 10 scientific research institutions is the same as that of the articles published. Among the institutions, Harvard University has been cited the most, with 43,578 citations. Literature published by University of Pennsylvania is the least cited, with a total of 15,810 citations. Both Harvard University and the University of California have been cited more than 40,000 times. It is reasonable to assume that these two institutions have the largest literature base with the highest quality in this field.

### Top 20 International Journals of Publications in Health Policy and Services

We rank journals based on Web of Science publications. According to Table 2, the top 20 journals of publications in health policy and services have included 28,941 papers, accounting for 49.8% of the total. The Journal Citation Report (JCR) published by Institute for Scientific Information (ISI) every year counts and calculates the citations and cited data between journals, and determines the journal's impact factor based on the average number of citations of the journal in 2 years. When the journal's impact factor is higher, the citation rate of its literature is also higher. To reflect the quality of a journal, all journals in a certain discipline are arranged in descending order according to the impact factor value of the previous year, and then divided into four quartiles: Q1, Q2, Q3, and Q4. According to JCR, and as seen in Table 2, when looking at journals covering health policy and services research, there were seven journals in Q1, seven in Q2, five in Q3, and one in Q4. The distribution indicates that the selected literature has a high impact factor and is at the leading level in the research field, which is of great significance for citation. The highest Web of Science publications is Health Affairs (2,523 articles) in Q1. AIDS Care-psychological and Socio-medical Aspects of AIDS/HIV in Q2 ranks the second with 2,288 articles. However, the citation frequency of AIDS Care-psychological and Socio-medical Aspects of AIDS/HIV (23,116 times) is significantly lower than that of Health Affairs (63,667 times).

**Table 2:** Journals of publications in health policy and services.

**Rank**	**Journal**	**Web of science publications**	**Citation frequency**	**Quartile**
1	Health Affairs	2,523	63,667	Q1
2	AIDS Care-psychological and Socio-medical Aspects of AIDS/HIV	2,288	23,116	Q2
3	Quality of Life Research	2,284	30,509	Q2
4	Psychiatric Services	1982	26,826	Q2
5	Health and Quality of Life Outcomes	1,690	20,132	Q2
6	Medical Care	1,689	30,552	Q1
7	Health Policy	1,506	14,808	Q2
8	American Journal of Managed Care	1,503	13,423	Q3
9	Value in Health	1,398	23,948	Q1
10	Journal of Community Health	1,327	9,196	Q3
11	Health Services Research	1,277	17,195	Q1
12	Health Economics	1,218	14,263	Q2
13	Implementation Science	1,216	27,309	Q1
14	Journal of Health Care for the Poor and Underserved	1,205	7,325	Q4
15	Health Communication	1,097	8,051	Q3
16	Community Mental Health Journal	1,067	6,812	Q3
17	Journal of Interprofessional Care	954	6,761	Q3
18	Health Policy and Planning	952	10,595	Q1
19	Journal of Genetic Counseling	898	6,651	Q2
20	Journal of Health Economics	867	15,514	Q1

*The ranking is in descending order of Web of Science publications*.

### Research Publications Across 10 Specific Academic Areas of Health Policy and Services

Research in the field of health policy and services covers a total of 92 specific disciplines, and the 10 specific disciplines with the largest number of papers (Table 3) are Health Policy & Services, Health Care Sciences & Services, Public, Environmental & Health, Economics, Social Sciences, Biomedical, Psychiatry, Psychology, unapproved, Respiratory System, Rehabilitation, Communication.

**Table 3:** Academic areas of health policy and services.

**Rank**	**Academic area**	**Number of publications**
1	Health Policy & Services	58,065
2	Health Care Sciences & Services	32,676
3	Public, Environmental & Occupational Health	19,921
4	Economics	5,874
5	Social Sciences, Biomedical	4,446
6	Psychiatry	3,343
7	Psychology, Multidisciplinary	2,660
8	Respiratory System	2,288
9	Rehabilitation	1,253
10	Communication	1,166

**Figure 3:**
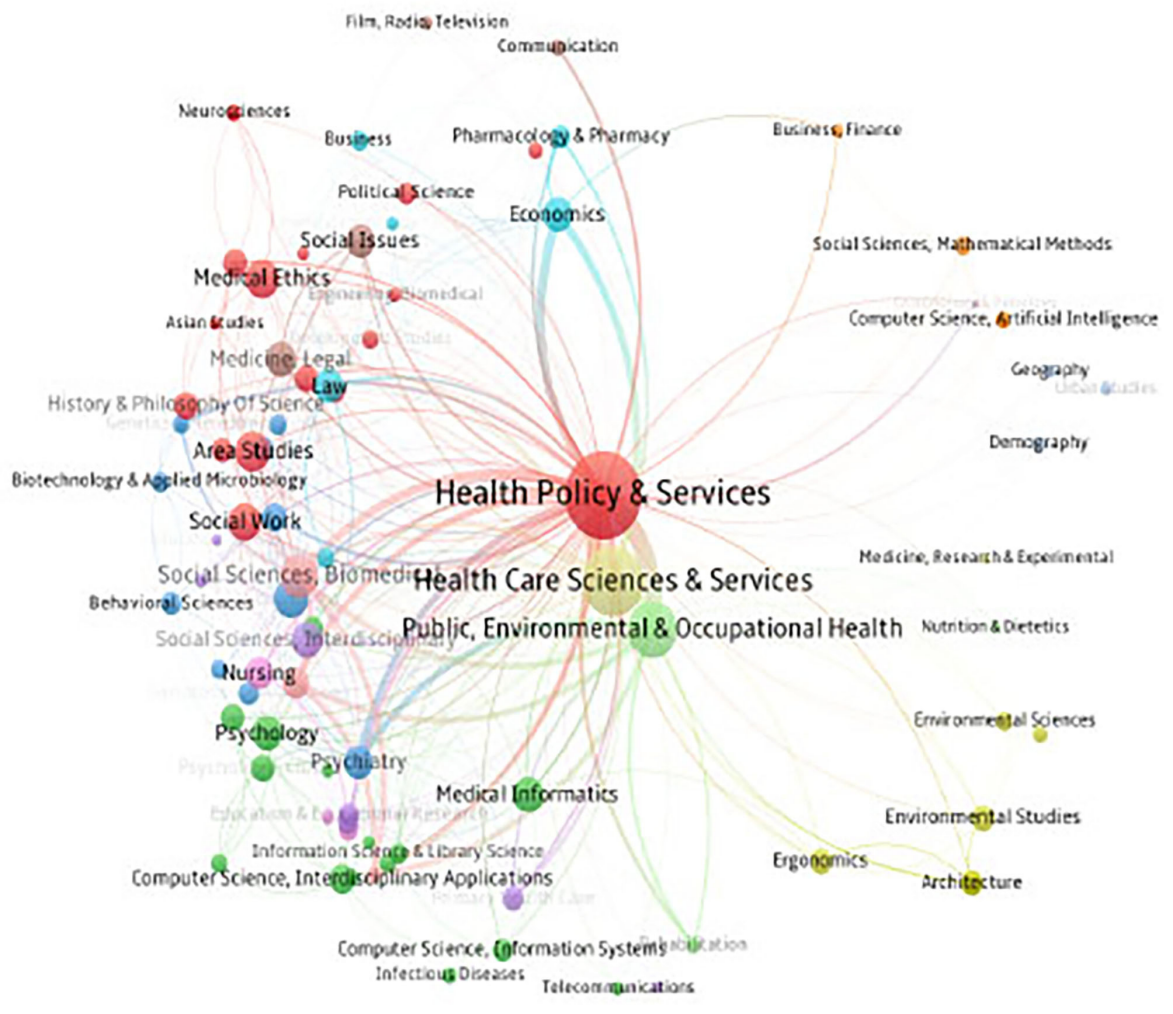
Academic areas covered by health policy and services.

As shown in Figure 3, through the co-journal approach, computing disciplines and structures related to health policy science form a skeleton diagram of disciplines related to health policy science. Co-journal refers to the division of the same journal based on the subject of the content. A journal may involve multiple subject categories, so a subject will have the same journal. Each node in the figure represents a discipline. The larger the node, the more papers published in the discipline. It can be seen that “Health Policy & Services,” “Health Care Science & Services,” “Public, Environmental & Occupational Health” are three major fields of health policy and services research. The connection between nodes indicates the correlation between disciplines. When there are more journals in common between disciplines, it is easier to form a closer relationship, and the discipline group formed between these disciplines is stronger. In Figure 3, the subject category “Health Policy & Services” is related to multiple subject categories in the circle. Among them, “Health Care Sciences & Services,” “Public, Environmental & Occupational Health,” “Economics,” “Social Science, Biomedical,” and “Psychiatry” are more closely related to it, which shows that they have more joint journals.

### Main Diseases, Research Targets, and Methods

As shown in Supplementary Table 1, most papers in the field of health policy and services focus on humans, such as “female,” “male,” “adult,” and “middle-aged.” They mainly use surveys and questionnaires, cross-sectional studies, qualitative research, interviews, and retrospective studies. Selecting the top 100 MeSH terms from these papers in the last 10 years can provide a good overview of the main diseases, research targets, and methods. According to the classification of the MeSH terms, common research targets include humans, female, male, adult, middle aged, aged, and the United States. Common research methods include surveys and questionnaires, cross-sectional studies, retrospective studies, and longitudinal studies. Common diseases include HIV infections, mental disorders, depression, physicians, and neoplasms.

Among the MeSH terms classified as research targets, “Human” has the largest number of records, reaching 17,194, followed by “Female” appearing 10,556 times. The MeSH term “surveys and questionnaires” is the most frequently used research method, with 3,581 records. Among the diseases, “HIV Infections” is the studied most often, with 1,067 records. Based on the number of MeSH terms recorded, the hotpots in the field of health policy and services from 2009 to 2018 are main factors related to disease or health in different age and gender groups, and the research on HIV infection. From the perspective of research content, literature on health assessment methods, health assessment research tools and standards of health assessment status have received the most attention. These studies include information on indicators for assessing health-related quality of life, innovations in the pharmaceutical industry, upgrading of direct measures, and the assessment of annual medical expenditures on obesity, all of which provide methodological support for health policy research.

### Recent Research Topics

**Figure 4:**
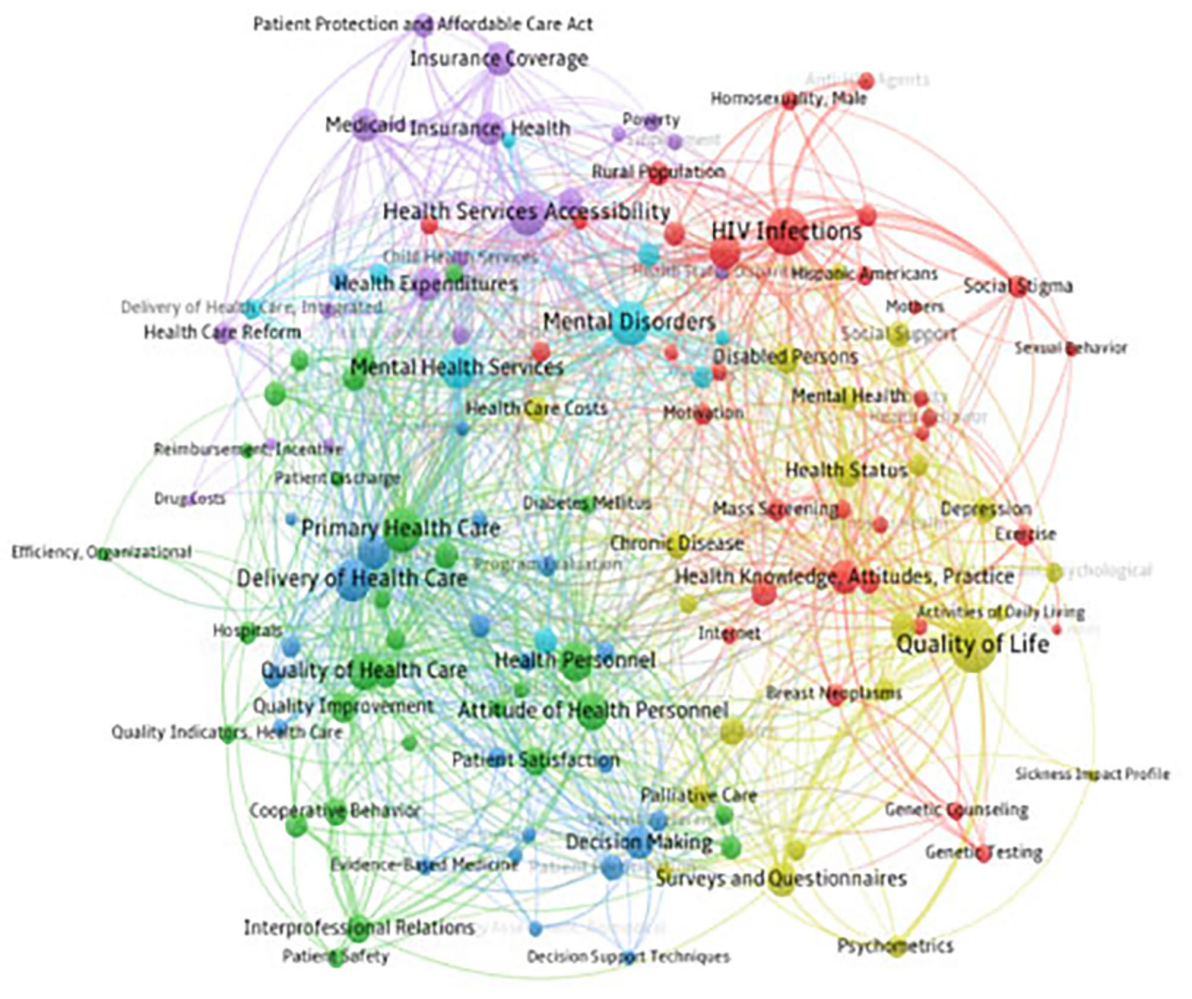
Research topics about health policy and services from 2016 to 2018.

This paper describes the research hotspots of health policy and services in the decade through the centrality of vocabulary. The clustering diagram of the co-occurrence of main keywords (Figure 4) shows the word frequency and centrality. The larger the dots, the higher the word frequency. The hot topics in the last 3 years are calculated based on 5,871 articles from 2016, 5,950 from 2017, and 6,329 from 2018.

**Figure 5:**
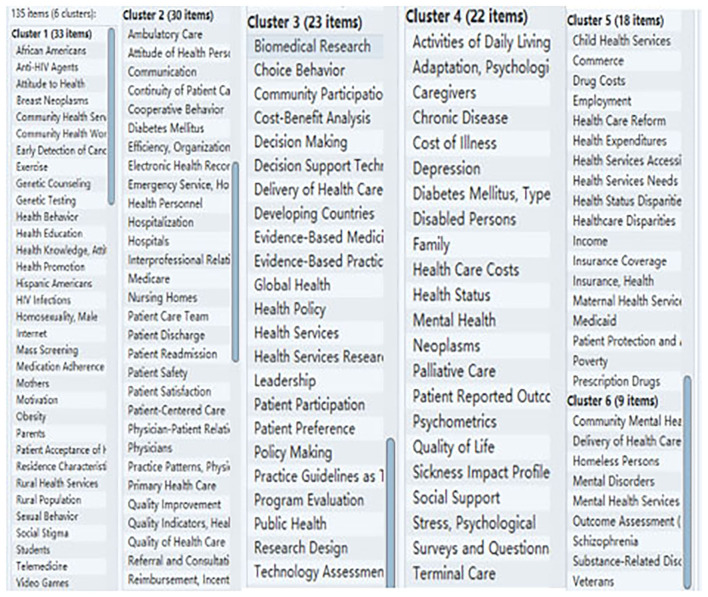
Clustering analysis results of health policy and services.

In 2016–2018, keywords with the highest word frequency are “HIV Infections,” “Primary Health Care,” “Delivery of Health Care,” and “Health Services Accessibility.” Cluster analysis was conducted on 135 high-frequency keywords divided into 6 categories was shown in Figure 5.

### Highly-Cited Papers on Health Policy and Services

As shown in Supplementary Table 2, highly-cited papers are another important aspect reflecting research hotspots. The research papers published in the field of health policy and services from 2009 to 2018 were ranked according to the Category Normalized Citation Impact (CNCI). The top 20 papers in terms of CNCI are shown in Supplementary Table 2. The paper with the highest CNCI is “Valuing health-related quality of life: An EQ-5D-5L value set for England” from Health Economics, cited 167 times by September 19, 2019 (CNCI = 142.0383). The top 20 papers are mainly from journals such as Health Economics and Implementation Science, of which 18 have been cited more than 200 times. The most cited paper is “Fostering implementation of health services research findings into practice: a consolidated framework for advancing implementation science” from Implementation Science, which has been cited 2,088 times and ranks second by CNCI (CNCI = 93.8544).

## Discussion

This bibliometric analysis provides an overview of the trends within health policy and services research from 2009 to 2018. For a long time, substantial effort has been devoted to catalyzing health policy and services research, but due to funding and resource limitations, some low and middle-income countries still have insufficient participation in this area (8). The article applies the bibliometric method to interpret the focus of health policy and service research in different countries and regions (especially China and the United States), the correlation between discipline categories, and the focus on diseases and research frontiers.

This article offers a unique contribution and builds upon the existing research on health policy and services using bibliometric analysis methods, in the following ways:

In terms of research scope, previous research in this area has been focused on a specific country or region. For example, Zyoud and colleagues used Microsoft Excel software to analyze the research productivity of toxicology in Middle Eastern Arab countries (9), and Sharma analyzed the published road traffic injury research in India (10). Comparatively, this article analyzes research productivity in the field of health policy and services based on a global perspective, so as to find research differences and changing trends between countries, and better grasp the frontiers of world research.

In terms of analysis indicators, previous studies utilize the basic indicators of bibliometric analysis. For example, Sweileh used basic indicators such as number and types of retrieved documents, author keywords, research domains, preferred journals, most active countries and journals to show the results of a bibliometric analysis of the global immigration health research in the peer-reviewed literature (11). In addition to the above basic indicators, this article also shows the main subject areas of the research, the cooperation of each subject area, and the results of cluster analysis of keywords. This helps to grasp the research hotspots and trends in the field of health policy and services.

At different times, different countries have different priorities for health policy and services. In last decade, the focus of this field has gradually shifted from public environmental occupational health (12–16), health policy services (17–21), and health science services (22–26) to specific diseases such as psychiatric (27–29) and pediatric diseases as well as environmental science ecology (30), biomedical social sciences and other fields. It has undergone a transformation from macro to micro. Generally, research on public environmental health, health policy services, and health science services are relatively mature. Developed countries are the main contributors to the research results in the field of health policy and services, and their research is more forward-looking, such as the research on the combination of pharmacology and health policy services. In addition to researching medical and health services and health care, the research on health policy and services also involves the fairness and accessibility of medical and health services, the reform of the medical and health system, traditional Chinese medicine, and infectious diseases like tuberculosis. However, in comparison, China's policy research on health care services is not as mature. All countries have strengths and weaknesses in their research, but have the opportunity to learn from one another. For example, learnings from the United States on pharmacology research can enhance traditional Chinese medicine. Additionally, China can learn from research in other countries on health services for pregnant women, newborns, and patients with mental health conditions.

Health Policy and Services research scientific policy has the characteristics of multi-disciplinary, prone to problem-solving, and obvious standardization. Figure 3 calculated by the method of co-journalism reflects the multidisciplinary nature of health policy and services research. Although the relationship between “health policy & services” and “economics,” “health care sciences & services,” “public,” “environmental & occupational health,” “social science,” “biomedical,” “psychiatry” and other disciplines is relatively close, there are still “geography,” “nutrition & dietetics,” “information science & library science” and other disciplines that are marginalized. It shows that health policy and service research should strengthen the correlation between economics, library science, information technology and science, journalism and communication, and enrich the research results in this field, and seek new breakthroughs. In the new post COVID 19 era, the rapid development of big data and artificial intelligence will lead to more precise, comprehensive, and rapid research. This will allow for improved conduction of empirical research on factors affecting population health, exploration of ways to formulate health policies in line with social development trends, and discovery of health policy and services breakthroughs in medical and health system quality improvement.

In terms of the diseases that are of major concern, in addition to the highly valued research on HIV/AIDS, mental disorders, chronic diseases, depression, and tumors have become the most concerning diseases in the health policy and services field during this period. According to a report released by The Joint United Nations Programme on HIV/AIDS(UNAIDS), as of 2018, the number of people living with HIV worldwide has reached 1.7 million, a decrease of 16% compared to 2010, which is mainly due to the progress made in combating HIV in southern and eastern African countries (31). This is consistent with the results of the literature measurement. Africa ranks third in the output of AIDS research in the field of health policy and services, after the United States and the United Kingdom. In China, according to the “China Health Statistics Yearbook” from 2009 to 2018, AIDS ranks first in the number of deaths from statutory infectious diseases reported in categories A and B, and its incidence has increased year by year. However, China's research results on fighting AIDS are very few, which points out a key area for China to pursue breakthrough research in. Additionally, in the past 10 years, the mortality rate of malignant tumors and the prevalence of chronic diseases have gradually increased in China. Similarly, China, Europe and the United States and other developed countries and regions on the relevant research for the prevention and treatment of cancer and chronic disease control obviously living in disadvantaged. Based on the high prevalence and high fatality rate of domestic infectious diseases, chronic diseases, malignant tumors, and depression, future research should focus on tackling these key diseases (32–41).

The co-occurrence cluster map of the main themes from 2016 to 2018 is further evidence of the above discussion. In the past 3 years, the focus of global attention in the field of health policy and services has alluded to the importance of fighting AIDS, enhancing the accessibility of medical and health services, the provision of medical and health services, and the importance of mental health services. These are the first issues that should be addressed in this field. China's focus is more on the reform of medical insurance (42, 43), the development and application of intelligent medical systems (44), and primary medical and health services. Western countries pay more attention to the application of mathematical-statistical analysis and other methods to verify or evaluate influencing factors. However, the application of this aspect is not limited to clinical disease causative factors, and environmental indicators are also taken into consideration.

It is found that although the total number of publications in the field of health policy and services in the US far exceeds that of China, the proportion of publications detailed in each research direction is different. For example, the proportion of publications in the research direction of Public Environmental Occupational Health in the US is 39.472%, while the proportion of publications in this research direction in China is 44.182%, which is nearly 5% higher than that in the US. Therefore, the US has an opportunity to learn from China. On the basis of maintaining extensive research on health policy and services, the US should strengthen its research in the following areas which currently represent less than half of the total amount of health policy and services research published to make up for the weaker aspects such as Public Environmental Occupational Health (accounting for 39.472%), Biomedical Social Sciences (accounting for 8.928%), Business Economics (accounting for 8.141%), Psychology (accounting for 5.755%), Respiratory System (accounting for 4.961%), Pharmacology Pharmacy (accounting for 1.078%), Government Law (accounting for 0.844%) and International Relations (accounting for 0.05%).

Similarly, compared with the US, China's research in the field of health policy and services needs to be strengthened. China has only 18 research directions, while the US has 21 research directions. In addition, China should also learn from the excellent research results of the US, and enhance the depth of research, especially for Psychiatry (accounting for 4.353%), Geriatrics Gerontology (accounting for 1.897%), Rehabilitation (accounting for 1.786%), Medical Informatics (accounting for 1.451%), to improve academic influence in the field. In general, while drawing on the experience of other regions and countries, China's health policy research should also incorporate the development status and characteristics of its own health system. Through a bibliometric analysis of global health policy and services research, this article shows the research trends in various countries and regions and highlights the areas that need to be further strengthened. The research directions of various countries and regions are basically the same for diseases that are showing a pandemic trend on a global scale. In addition, the focus of each country and region is different according to its own context. For example, the research focus of China tends to the goal of medical and health system reform.

### Strengths and Limitations

Using bibliometrics methods to analyze research in the field of health policy and services, we can directly understand, the scientific research contributions made by countries and regions to the development of the field, the scientific research institutions and journals, research hotspots and main research directions. Through this research method, you can quickly see the research progress of different countries and regions in the field of health policy and services, find research gaps, and help to formulate health policies and provide health services in a targeted manner.

Despite these strengths, there are some limitations to be acknowledged. This study is just based on the Web of Science database, although we believe it fully depicts the research results of the past 10 years. However, our findings may not necessarily represent the overall development of the health policy and services field beyond this what is provided in this database and the specified timeframe. In addition, as mentioned previously in this article, COVID-19 related articles were not included in the study. The inclusion of these articles had the potential of skewing our findings and masking identified trends, but we are considering conducting a comprehensive and in-depth review and analysis on COVID-19 in the future.

## Conclusion

Studying journal articles on health policy and services in the Web of Science database over the past decade, we have found that scholars and experts in this field are working on the most effective ways to improve people's health and living standards. The methods and indicators of health evaluation have become the focus of research, which is also the general trend of China's health policy research. Highly cited papers have important reference value for discussion on research methods, standards, and models. Statistical analysis of the Web of Science database indicates that most papers are from developed regions and countries such as Europe and the US, and the most influential research institutions and journals are also mainly from the UK and the US. In terms of the number of publications in the field of health policy and services, China should learn from developed countries to improve research productivity.

## Data Availability Statement

The original contributions presented in the study are included in the article/Supplementary Material, further inquiries can be directed to the corresponding author/s.

## Author Contributions

GC conceived and designed the study. LZ, JD, and GC did the initial analysis and supervised data analysis. LZ and YZ wrote the first draft of the paper. YZ, AD, ZH, and GC critically revised the first draft. All authors reviewed and approved the final version of the paper submitted for publication.

## Funding

The National Social Science Fund of China (81602869) was funded by National Office of Philosophy and Social Science.

## Conflict of Interest

The authors declare that the research was conducted in the absence of any commercial or financial relationships that could be construed as a potential conflict of interest.

## Publisher's Note

All claims expressed in this article are solely those of the authors and do not necessarily represent those of their affiliated organizations, or those of the publisher, the editors and the reviewers. Any product that may be evaluated in this article, or claim that may be made by its manufacturer, is not guaranteed or endorsed by the publisher.
