# Supplementary material for: Mapping the Research on Health Policy and Services in the Last Decade (2009–2018): A Bibliometric Analysis
Source: Front Public Health. 2022 Apr 27;10:773668. doi: 10.3389/fpubh.2022.773668 (PMC9092023; doi:10.3389/fpubh.2022.773668)
Supplement: Supplementary file 1 [file Data_Sheet_1.pdf]

Supplementary Material

1 Supplementary Tables

Supplementary Table 1 The top 100 MeSH Terms of health policy and services.

	MeSH terms (top-level)	Number of		MeSH terms (top-level)	Number of
1	Humans	17194	51	Pregnancy	539
2	Female	10556	52	Time Factors	539
3	Male	9724	53	Medicaid	532
4	Adult	6847	54	Social Support	515
5	Middle Aged	6308	55	Program Evaluation	507
6	Aged	4266	56	Health Promotion	506
7	Surveys and	3581	57	Rural Population	499
8	United States	3356	58	Chronic Disease	498
9	Adolescent	2898	59	Prospective Studies	498
10	Young Adult	2823	60	Patient Satisfaction	497
11	Cross-Sectional Studies	1991	61	Health Care Costs	495
12	Quality of Life	1974	62	Depression	489
13	Aged, 80 and over	1758	63	Logistic Models	486
14	Child	1502	64	Physicians	484
15	Qualitative Research	1461	65	Insurance Coverage	478
16	Socioeconomic Factors	1117	66	Hospitals	448
17	Interviews as Topic	1078	67	Mental Health	438
18	HIV Infections	1067	68	Neoplasms	436
19	Delivery of Health Care	1063	69	Outcome Assessment (Health	434
20	Health Services	1053	70	Prevalence	433
21	Retrospective Studies	993	71	Cohort Studies	421
22	Health Policy	986	72	Interprofessional Relations	417
23	Primary Health Care	970	73	Caregivers	415
24	Attitude of Health	876	74	Poverty	411
25	Quality of Health Care	864	75	Treatment Outcome	406
26	Mental Disorders	849	76	Health Services Research	398
27	Cost-Benefit Analysis	795	77	Disabled Persons	396
28	Health Knowledge,	793	78	Patient Care Team	395
29	Health Status	791	79	Health Surveys	385
30	Reproducibility of	772	80	Parents	383
31	Health Personnel	757	81	Databases, Factual	382
32	Health Expenditures	745	82	United Kingdom	382
33	Risk Factors	738	83	Infant, Newborn	381
34	Decision Making	728	84	Emergency Service, Hospital	377
35	Child, Preschool	693	85	Patient Safety	372
36	Quality Improvement	678	86	Patient Protection and	369
37	Age Factors	638	87	Patient-Centered Care	366
38	Psychometrics	617	88	Developing Countries	358
39	Insurance, Health	608	89	Quality-Adjusted Life Years	355
40	Mental Health Services	592	90	Palliative Care	351
41	Cooperative Behavior	578	91	Health Care Reform	350
42	Australia	561	92	China	349
43	Longitudinal Studies	556	93	Healthcare Disparities	349
44	Sex Factors	556	94	Self Report	344
45	Medicare	553	95	Public Health	340
46	Focus Groups	549	96	Pilot Projects	334
47	Communication	547	97	Motivation	332

48	Hospitalization	544	98	Internet	331
49	Infant	541	99	Substance-Related Disorders	326
50	Patient Acceptance of	539	100	Patient Participation	318

Supplementary Table 2 Highly cited papers on health policy and services.

	**Title**	**Source**	**Publication year**	**Citation frequency**	**CNCI**
1	Valuing health-related quality of life: An EQ-5D-5L value set for England	HEALTH ECONOMICS	2018	167	142.0383
2	Fostering implementation of health services research findings into practice: a consolidated framework for advancing implementation science	IMPLEMENTATION SCIENCE	2009	2088	93.8544
3	Innovation in the pharmaceutical industry: New estimates of R&D costs	JOURNAL OF HEALTH ECONOMICS	2016	413	83.686
4	The behaviour change wheel: A new method for characterising and designing behaviour change interventions	IMPLEMENTATION SCIENCE	2011	1276	77.7861
5	Development and preliminary testing of the new five-level version of EQ-5D (EQ-5D-5L)	QUALITY OF LIFE RESEARCH	2011	1210	73.7627
6	Scoping studies: advancing the methodology	IMPLEMENTATION SCIENCE	2010	1331	70.4862
7	Making sense of implementation theories, models and frameworks	IMPLEMENTATION SCIENCE	2015	437	64.3785
8	Purposeful Sampling for Qualitative Data Collection and Analysis in Mixed Method Implementation Research	ADMINISTRATION AND POLICY IN MENTAL HEALTH AND MENTAL HEALTH SERVICES RESEARCH	2015	434	63.9365
9	The COSMIN checklist for assessing the methodological quality of studies on measurement properties of health status measurement instruments: an international Delphi study	QUALITY OF LIFE RESEARCH	2010	985	52.1629
10	Annual Medical Spending Attributable To Obesity: Payer-And Service-Specific Estimates	HEALTH AFFAIRS	2009	1154	51.8717
11	Validation of the theoretical domains framework for use in behaviour change and implementation research	IMPLEMENTATION SCIENCE	2012	663	47.3055
12	Knowledge translation of research findings	IMPLEMENTATION SCIENCE	2012	651	46.4493
13	Disrespectful and abusive treatment during facility delivery in Tanzania: a facility and community survey	HEALTH POLICY AND PLANNING	2018	53	45.078
14	Consolidated Health Economic Evaluation Reporting Standards (CHEERS)-Explanation and Elaboration: A Report of the ISPOR Health Economic Evaluation Publication Guidelines Good Reporting Practices Task Force	VALUE IN HEALTH	2013	513	44.6529
15	Outcomes for Implementation Research: Conceptual Distinctions, Measurement Challenges, and Research Agenda	ADMINISTRATION AND POLICY IN MENTAL HEALTH AND MENTAL HEALTH SERVICES RESEARCH	2011	723	44.0747
16	Advancing a Conceptual Model of Evidence-Based Practice Implementation in Public Service Sectors	ADMINISTRATION AND POLICY IN MENTAL HEALTH AND MENTAL HEALTH SERVICES RESEARCH	2011	683	41.6363
17	What The Evidence Shows About Patient Activation: Better Health Outcomes And Care Experiences; Fewer Data On Costs	HEALTH AFFAIRS	2013	475	41.3452
18	A refined compilation of implementation strategies: results from the Expert Recommendations for Implementing Change (ERIC) project	IMPLEMENTATION SCIENCE	2015	266	39.1869
19	The medical care costs of obesity: An instrumental variables approach	JOURNAL OF HEALTH ECONOMICS	2012	548	39.1002
20	The Mass Production of Redundant, Misleading, and Conflicted Systematic Reviews and Meta-analyses	MILBANK QUARTERLY	2016	185	37.4865
